# A multimethod longitudinal examination of the effects of childhood maltreatment on birth experiences and postpartum mental health

**DOI:** 10.1017/S0954579425100369

**Published:** 2025-08-04

**Authors:** Kira R. Wright, Anna M. Zhou, Nicolette C. Molina, Nina de Vos, Parisa R. Kaliush, Elisabeth Conradt, Sheila E. Crowell

**Affiliations:** 1 Department of Psychology, University of Utah, Salt Lake City, UT, USA,; 2 Department of Psychiatry, University of Colorado Anschutz Medical Campus, Aurora, CO, USA,; 3 Department of Psychology, University of Denver, Denver, CO, USA,; 4 Department of Psychology, University of Oregon, Eugene, OR, USA,; 5 Department of Psychiatry, University of North Carolina at Chapel Hill School of Medicine, Chapel Hill, NC, USA; 6 Department of Psychiatry and Behavioral Sciences, Duke University School of Medicine, Durham, NC, USA

**Keywords:** Birth experiences, childhood maltreatment, emotion dysregulation, maternal mental health, respiratory sinus arrhythmia

## Abstract

The perinatal period has gained increasing attention from developmental psychopathologists; however, experiences during birth have been minimally examined using this framework. The current study aimed to evaluate longitudinal associations between childhood maltreatment, negative birth experiences, and postpartum mental health across levels of self-reported emotion dysregulation and respiratory sinus arrhythmia (RSA). Expectant mothers (*N* = 223) participated in a longitudinal study from the third trimester of pregnancy to 7 months postpartum. Participants contributed prenatal resting RSA and completed questionnaires prenatally, 24 hours after birth, and 7 months postpartum. Results indicated that more childhood maltreatment was associated with higher birth fear and postpartum anxiety and depressive symptoms. Resting RSA moderated the association between childhood maltreatment and birth fear, such that more childhood maltreatment and higher resting RSA were associated with increased birth fear. Additionally, self-reported prenatal emotion dysregulation moderated the association between childhood maltreatment and postpartum depressive symptoms, such that more childhood maltreatment and higher emotion dysregulation were associated with increased depressive symptoms. Emotion dysregulation across multiple levels may amplify vulnerability to negative birth experiences and postpartum psychopathology among individuals with childhood maltreatment histories. Thus, emotion dysregulation in the context of trauma-informed care may be worthwhile intervention targets during the perinatal period.

## Introduction

One in seven birthing parents report symptoms of postpartum depression within the first year after birth (Carlson et al., 2024), with comorbid depression and anxiety occurring in about 13% of birthing parents postpartum (Falah-Hassani et al., 2016; Shen et al., 2024). Given the range of psychological and physiological changes experienced during the perinatal period, scholars examining this period use a developmental psychopathology framework and posit that it is a sensitive period of development that has important implications for maternal mental health (Davis & Narayan, 2020; Glynn et al., 2018; Goodman & Dimidjian, 2012; Howard & Khalifeh, 2020). The developmental psychopathology framework emphasizes longitudinal, multimethod investigations to evaluate dynamic adaptive and maladaptive health outcomes during periods of development across the lifespan (Cicchetti & Rogosch, 1996; Cicchetti & Toth, 2009). This framework offers a lens to examine the intersection of psychological, physiological, and environmental changes during the transition to parenthood as potential mechanisms underlying changes in mental health outcomes across the perinatal period (Doyle & Cicchetti, 2018).

Despite increasing attention to the perinatal period among developmental psychopathologists, birth, a monumental psychophysiological event, remains largely understudied in the literature on perinatal mental health (Kaliush et al., 2023a). This oversight is significant, as negative birth experiences can have implications for a vast array of postpartum health outcomes (for reviews, see Kaliush et al., 2023a; Simpson & Catling, 2016). Negative emotional experiences during birth may be a pathway by which earlier risk factors contribute to symptoms of postpartum depression and anxiety (e.g., Ahmadpour et al., 2023). Therefore, a developmental perspective is needed to examine the role of birth experiences and perinatal mental health during a significant transitionary period. A recent developmental psychopathology model of the perinatal period highlighted how birth experiences critically bridge prenatal and postnatal health (Kaliush et al., 2023a). We apply and extend this framework in the current study to examine how earlier risk factors – childhood maltreatment, a historical factor, and prenatal emotion dysregulation, a factor closer in time to birth – may be associated with negative birthing experiences, as well as maternal anxiety and depressive symptoms at 7 months postpartum.

### The birth experience and postpartum mental health outcomes

The birth experience is an important predictor of postpartum mental health outcomes. During this time, birthing parents undergo biological, psychological, and sociocultural changes that dynamically interact to contribute to postpartum outcomes (Kaliush et al., 2023a). Negative aspects of the birth experience, such as fear, stress, and trauma, can have adverse influences on postpartum outcomes for both mother and infant. Perceiving the birth experience as negative has been associated with increased symptoms of postpartum depression (Ahmadpour et al., 2023; Bell & Andersson, 2016) and an increase in anxiety symptoms (Fallon et al., 2022; Fenech & Thomson, 2014). Additionally, negative birth experiences have been associated with relationship difficulties, emotional turmoil, and symptoms of PTSD (Bell & Andersson, 2016; Cigoli et al., 2006; Fenech & Thomson, 2014). Eitenmuller et al. (2022) found postpartum depression facilitated the relation between negative emotional experiences during birth and lower quality mother-infant bonding postpartum. Similarly, another study found postpartum PTSD symptoms to be associated with poorer child socioemotional outcomes two years after birth (Garthus-Niegal et al., 2017).

Perceptions of a negative birth experience can therefore impact maternal postpartum well-being and, in turn, have implications for infant outcomes (Mcdonnell & Valentino, 2016; Seng et al., 2013). Yet, current literature has minimally examined specific facets of the birth experience, such as fear and stress, in relation to postpartum mental health outcomes. Various studies have identified social support, quality of care during birth, and unexpected birth complications as contributing to a negative birth experience and influencing postpartum mental health outcomes (Cárdenas et al., 2025; Cigoli et al., 2006; Fallon et al., 2022; Karakoç et al., 2023). While some work has begun to uncover related factors, subjective facets (e.g., perceptions of fear and stress) of the birth experience have been overlooked in relation to postpartum mental health. Early-life and prenatal risk factors may increase the likelihood of perceiving birth as a negative experience, which can impact birthing parents’ postpartum mental health. One study identified a positive association between birthing parents’ existing fear of birth and their perception of a negative birth experience, and in turn negative birth experiences were associated with worsened mother-infant bonding (Seefeld et al., 2022). Together, this literature suggests that longitudinal examinations over the duration of the perinatal period are needed to identify the potential risk factors that may influence perception of the birth experience and postpartum mental health outcomes.

### Maternal childhood maltreatment and the perinatal period

Several studies indicate that birthing parents with histories of childhood maltreatment or adverse childhood experiences report more symptoms of depression, PTSD, and self-injurious thoughts postpartum (Fekih-Romdhane et al., 2024; Mcdonnell & Valentino, 2016; Molina et al., 2025; Osofsky et al., 2021). However, not all findings are consistent; for example, Lev-Wiesel et al. (2009) found no associations between histories of childhood sexual abuse and PTSD symptoms at 2- and 6-months postpartum. Similarly, Choi and Sikkema (2016) identified trends in the literature and noted that while childhood maltreatment is associated with perinatal depression and PTSD, findings for postpartum anxiety were inconsistent. The authors theorized that anxiety symptoms may be common across the perinatal period and not significantly influenced by experiences of trauma in perinatal populations (Choi & Sikkema, 2016). Further research is needed to clarify the underlying factors involved in these associations.

Birthing parents with histories of childhood maltreatment have also been shown to report more negative birth experiences. Specifically, birthing parents with a history of childhood sexual abuse reported their birth experience as being more frightening and experienced an increase in intrusive thoughts and arousal during birth (Leeners et al., 2016; Lev-Wiesel et al., 2009). Similarly, Porthan et al. (2023) found that childhood emotional abuse and neglect were associated with increased fear during birth. Previous literature has examined individual components of childhood maltreatment in relation to the birth experience and postpartum outcomes (i.e. sexual abuse, emotional neglect, or emotional abuse), but has minimally examined overall childhood maltreatment accumulation despite the co-occurrence of complex trauma experiences during childhood (Cruz et al., 2022). Capturing shared rather than distinct experiences of childhood maltreatment may better inform transdiagnostic interventions for high-risk populations. These transdiagnostic interventions could also address transdiagnostic mental health symptoms by targeting core mechanisms of emotion dysregulation, such as distress tolerance, that might contribute to birth-specific fear.

### The role of prenatal emotion dysregulation

Emotion dysregulation refers to difficulty with monitoring, evaluating, and modifying emotional responses and is marked by high sensitivity to emotion stimulating situations (Crowell et al., 2013; Gratz & Roemer, 2004). Furthermore, emotion dysregulation is a known transdiagnostic risk factor for psychopathology (Cole & Hall, 2008; Cuthbert, 2020; Fernandez et al., 2016). More severe childhood maltreatment has been associated with higher emotion dysregulation during pregnancy (Kaliush et al., 2021). In addition, many studies have shown that emotion dysregulation may exacerbate associations between childhood maltreatment and long-term mental health outcomes and have found associations between childhood maltreatment, emotion dysregulation, and later psychopathology (for review, see Dvir et al., 2014). Therefore, emotion dysregulation may strengthen the association between childhood maltreatment and perceptions of fear and stress during birth experiences. For example, Compton et al. (2023) found that emotion dysregulation influenced the association between positive memory recall and hazardous substance use in populations that experienced childhood maltreatment, suggesting that emotion dysregulation can have a significant influence on long-term outcomes among populations with childhood maltreatment histories. Hence, prenatal maternal emotion dysregulation may modify associations between childhood maltreatment and birth experiences; however, this has not been explored previously.

Few studies have examined physiological measures in relation to birth experiences as well. With birth being an arousing physiological experience, it is important to consider how individual differences in physiological regulation may influence the associations between childhood maltreatment, birth experiences, and postpartum mental health. Individual variations in physiological responsivity during the perinatal period may contribute to emotional experiences during birth and postpartum health outcomes (Kaliush et al., 2023a). Specifically, respiratory sinus arrhythmia (RSA) is a measure of high-frequency variability in heart rate across the respiratory cycle and an index of parasympathetic nervous system activity (Beauchaine & Thayer, 2015). Resting RSA reflects the ability to adapt to environmental demands. Individuals with higher resting RSA are often able to more efficiently regulate their emotional arousal, which is indicative of a greater capacity for emotion regulation (Beauchaine, 2015). In contrast, lower resting RSA is often associated with increased vulnerability for emotion dysregulation (Beauchaine, 2015). Although higher resting RSA is often conceptualized as a marker of regulatory capacity (Beauchaine & Thayer, 2015), there is literature to suggest it may also reflect heightened sensitivity to context. Theories of biological sensitivity to context posit that individuals with higher physiological reactivity may be more responsive to both supportive and stressful environments (Boyce & Ellis, 2005). In the context of a highly stressful experience like birth, it may be that individuals with higher RSA are more attuned to physiological cues, which in turn may heighten fear when childbirth is perceived as threatening. Thus, high RSA may function as a protective factor or a marker of plasticity, depending on the individual’s prior experiences (e.g., childhood maltreatment) and interpretation of birthing contexts. The current study examines whether prenatal RSA moderates the association between childhood maltreatment trauma and emotional experiences during birth, allowing us to test these alternative theoretical interpretations.

Previous literature has shown evidence of links between maternal RSA during the perinatal period and maternal mental health (Brandes-Aitken et al., 2024; Lin et al., 2019; Singh Solorzano et al., 2022; Zhou et al., 2024). However, prior work has shown that there were no direct associations between experiences of childhood maltreatment and prenatal maternal heart rate variability (Kaliush et al., 2021). Despite the lack of direct associations in prior literature, the interaction between childhood maltreatment and prenatal RSA may modify the relation between earlier risk factors and the birth experience as well as the relation with postpartum mental health. Capturing various indicators of emotion dysregulation (i.e., self-report and RSA) may better disentangle the current mixed findings in the literature. Multimethod assessments of emotion dysregulation can help us better characterize the psychobiological processes that may influence associations between childhood maltreatment and perinatal outcomes.

### The present study

The current longitudinal study examined how childhood maltreatment accumulation and prenatal emotion dysregulation were associated with negative emotional experiences during birth (e.g., fear and stress) and postpartum mental health (e.g., anxiety and depressive symptoms). Extant literature has overlooked how subjective birth experiences relate to long-term postpartum outcomes. Similarly, specific components of childhood maltreatment have been examined in relation to birth experiences, but this approach overlooks the co-occurrence of multiple types of trauma experiences in childhood. In addition, there has been minimal examination of birth experiences as a potential mediator of associations between childhood maltreatment and postpartum mental health during the perinatal period despite past findings that birth experiences mediate associations between prenatal birth fear and mother-infant bonding postpartum (Seefeld et al., 2022).

**Figure 1.**
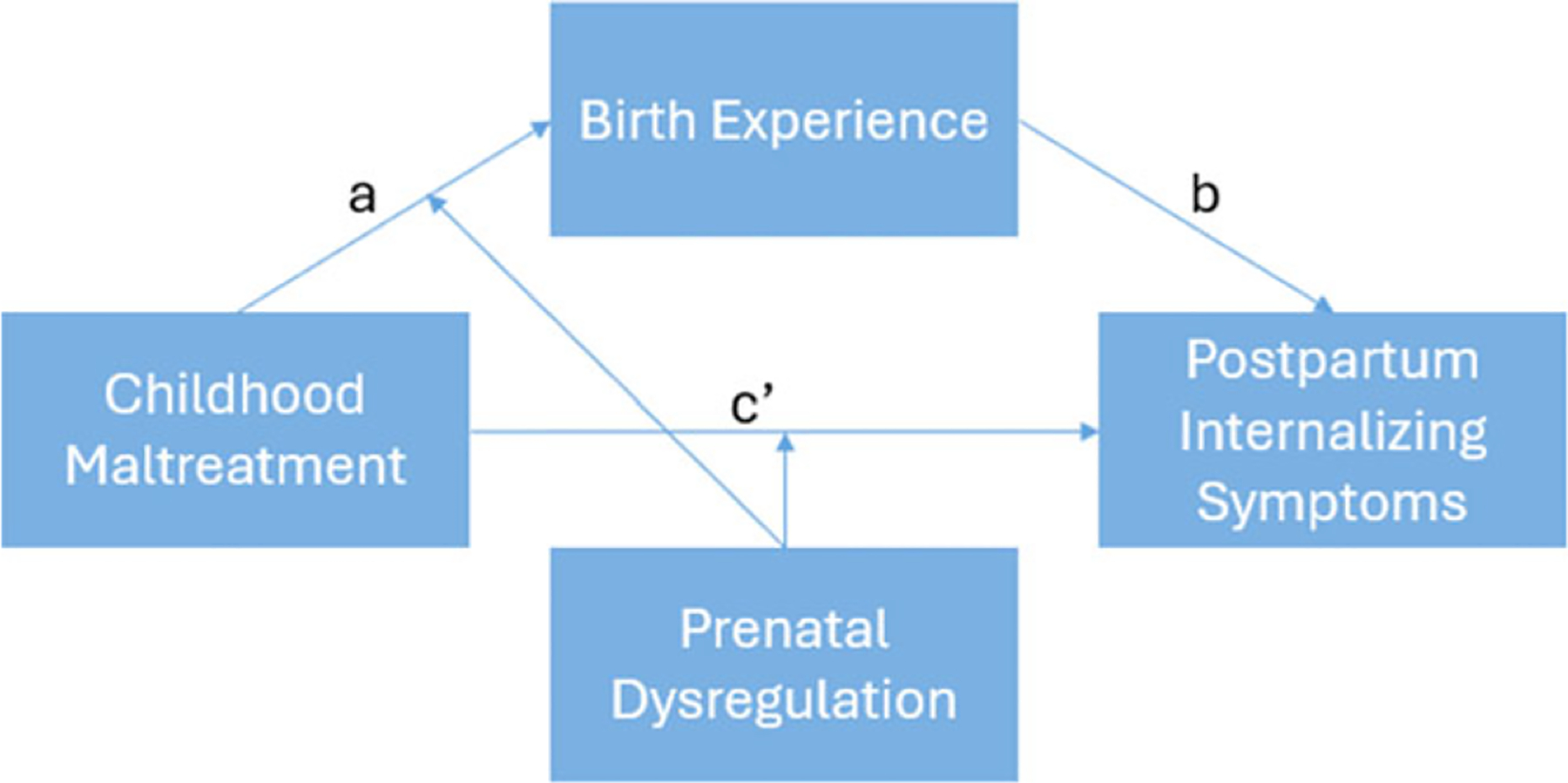
Conceptual model displaying the longitudinal paths examined between childhood maltreatment, negative emotional experiences during birth, and postpartum mental health, as well as the interaction between childhood maltreatment and emotion dysregulation.

Finally, mixed findings in the literature may be due to unexplored moderators of the pathways between childhood maltreatment, birth experiences, and postpartum mental health. Due to significant relations between emotion dysregulation and childhood maltreatment, as well as the transdiagnostic role of emotion dysregulation underlying many mental health conditions, emotion dysregulation may strengthen the anticipated associations in this study. Thus, we examined prenatal self-reported emotion dysregulation and prenatal maternal resting RSA, a physiological measure of emotion regulation capacity (Beauchaine & Thayer, 2015), as moderators of the relation between childhood maltreatment, birth experiences, and postpartum mental health. Figure 1 depicts the conceptual model for this study.

We predicted that for birthing parents who report higher emotion dysregulation and have lower resting RSA, higher reported childhood maltreatment would be associated with greater fear and stress during birth. Lower emotion dysregulation and higher resting RSA during pregnancy may buffer the association between past maltreatment and birth experiences. Additionally, we examined associations between negative emotions during birth and maternal mental health outcomes 7 months postpartum. We predicted that higher fear and stress during the birth experience would be associated with increased anxiety and depressive symptoms postpartum. Lastly, we examined the mediating role of birth emotions in the association between childhood maltreatment and postpartum mental health outcomes. We predicted a positive indirect association between childhood maltreatment and maternal mental health postpartum when there are greater reports of fear and stress during birth.

## Method

### Participants

The sample consists of 223 mothers from a larger longitudinal study examining intergenerational transmission of emotion dysregulation from the 3^rd^ trimester of pregnancy to 36 months postpartum. Birthing parents were recruited from local obstetric clinics, via email, and advertisements in the community from 2019 to 2023. During recruitment, participants completed a screening measure consisting of eligibility criteria questions (i.e. age 18–40, no pregnancy complications, no substance use, and anticipation of a singleton delivery) and the Difficulties in Emotion Regulation Scale (DERS; Gratz & Roemer, 2004). The larger study over-sampled for high and low levels of emotion dysregulation to achieve a uniform distribution of DERS scores. Within the sample, 59.2% of participants identified as White and Not Hispanic/Latina, a third obtained a four-year college degree (31.8%), almost a quarter reported having a household income of $50,000 to $79,999 (22.0%) and another quarter reported $100,000 or more (23.8%). Additionally, the majority of participants were married at the prenatal timepoint (83.9%). Detailed demographic information for the sample is presented in Table 1.

### Measures

#### Traumatic experiences of betrayal across the lifespan

The Traumatic Experiences of Betrayal Across the Lifespan (TEBL; Kaliush et al., 2023b) was administered to assess lifetime trauma. The TEBL consists of 22 yes/no items that retrospectively evaluate traumatic experiences, including childhood maltreatment. Participants reported the frequency of specific maltreatment experiences and the age range at which each occurred with prompts such as “At any time in your life, has someone with whom you were close or trusted … ” and “approximately how many times” the event occurred. For this study, we focused on events that occurred by age 17 to capture childhood maltreatment. Six items reflecting five experiences – physical abuse, physical neglect, emotional abuse, emotional neglect, and sexual abuse – were selected, consistent with validated approaches (e.g., Childhood Trauma Questionnaire; Bernstein et al., 2003). A cumulative maltreatment score was calculated based on the endorsement of these experiences. The TEBL showed good reliability (*r* = .83) and good internal consistency (Cronbach’s *a* = .80) for our sample. For more details on measure items from the TEBL included in analyses, see Table S1 in supplemental materials. Additional information regarding maltreatment co-occurrence and maltreatment subtype endorsement for our sample is presented in Table S2 and Figure S1, respectively, in supplemental materials.

#### Difficulties in emotion regulation scale

The Difficulties in Emotion Regulation Scale (DERS; Gratz & Roemer, 2004) was administered to measure prenatal maternal emotion dysregulation during the 3^rd^ trimester of pregnancy. The DERS consists of 36 items to measure maternal emotion dysregulation and uses a 5-point Likert scale (1- *almost never*, 5- *almost always*). Six aspects of emotion dysregulation were assessed: nonacceptance of emotional responses, difficulty engaging in goal directed behavior, difficulties with impulse control, lack of emotional awareness, lack of emotional clarity, and limited access to emotion regulation strategies. Participant responses were summed to generate an overall emotion dysregulation score, where larger values represent greater levels of emotion dysregulation. The DERS demonstrated strong internal consistency for the current sample (Cronbach’s *a* = .96).

#### Prenatal resting RSA

Maternal electrocardiogram (ECG) data were collected during the prenatal lab visit using Mindware mobile devices (Mindware Technologies Ltd). In the lab, trained staff placed electrodes on the participants’ chest and torso based on a three-lead configuration. During the COVID-19 pandemic, remote research sessions were conducted in the participants’ home, and participants placed the electrodes themselves while being guided by trained staff remotely (see Gao et al., 2022 for remote research visit protocol). Mindware Technologies HRV 3.1.5 software was used to collect and process ECG data. RSA data were extracted from ECG data in 60-second epochs for a total of 10 minutes, during which the mother was instructed to relax and not engage in any activities to establish a baseline. RSA was processed as the beat-to-beat variability in ECG R-peaks in the high frequency band of .24–.60 Hz due to high respiration levels observed among pregnant populations. Resting RSA was calculated as the average RSA across the 10-minute baseline task.

### Birth experiences questionnaire

The Birth Experiences Questionnaire (BEQ); Saxbe et al. (2018) is a 10 item self-report measure administered shortly after birth that is designed to assess stress, fear, and support during the birth experience. The BEQ is scored on a 7-point Likert scale (1- *not at all*, 7- *extremely)* based on how the participants found the birth experience (e.g., *Did you fear for your life?*) with an additional item asking to rate their overall birth experience from 1 (*Extremely negative*) to 7 (*Extremely Positive*). A confirmatory factor analysis (CFA), based on the three-factor solution in Saxbe et al. (2018), showed that a three-factor solution fit our data well, χ^2^ = 47.26, *p* = .003, CFI = .95, TLI = .92, RMSEA = .07, SRMR = .05. Of particular interest to the current study, the subscales for stress and fear were used to examine negative emotions associated with the birth experience. Internal consistency for the stress subscale was acceptable (*α* = .80). However, internal consistency for the fear subscale was lower (*α* = .57), although it should be noted that Cronbach’s alpha is not appropriate for scales with two items (Eisinga et al., 2013). Given that the two items for fear significantly loaded onto a single factor in the CFA and replicated prior factor analyses, we proceeded to utilize this subscale. Mean scores were computed for items belonging to each subscale, with higher scores indicating more stress or fear during the birth experience.

#### Achenbach system of empirically based assessment adult self report

Postpartum anxiety and depressive symptoms were assessed with the Achenbach System of Empirically Based Assessment Adult Self Report (ASEBA-ASR; Achenbach et al., 2003), which examines behavioral, emotional, social, and clinical problems for adults. The ASEBA-ASR consists of 183 items on a 3- point Likert scale (0- *not true*, 2-*very true*). Various subscales are computed from this measure but of particular interest to the current study are the DSM-oriented subscales for depressive problems and anxiety problems. The DSM-oriented subscale for depressive symptoms consists of 14 items such as “*I cry a lot*” and “*I am unhappy, sad, or depressed*.” The DSM-oriented subscale for anxiety symptoms consists of 7 items such as “*I worry about my future*” and “*I am afraid of certain animals, situations, or places*.” The subscales for depressive and anxiety problems were summed to compute a total score with a higher score indicating higher instances of depressive or anxiety problems. Each subscale showed good internal consistency for our sample (depressive problems Cronbach’s *a* = .86; anxiety problems Cronbach’s *a* = .81).

### Procedures

Upon consenting to participate in the larger longitudinal study, eligible participants completed questionnaires evaluating experiences of childhood maltreatment accumulation (TEBL), prenatal emotion dysregulation (DERS), and demographic information using an online link. At the prenatal timepoint, expectant mothers contributed resting RSA during a 10-minute rest period prior to participating in behavioral tasks during which physiological data were collected (for additional details regarding remote research visits during the COVID-19 pandemic, see Gao et al., 2022). 24 hours after delivery, participants completed the birth experience questionnaire (BEQ) using an online link. Prior to completing the 7 month lab visit, mothers completed online questionnaires about their postpartum mental health, specifically anxiety and depressive symptoms. Study procedures were approved by the Institutional Review Board at the University of Utah.

### Data analytic plan

We conducted path models in Mplus 8.10 (Muthén & Muthén, 2010) to test if childhood maltreatment, prenatal maternal emotion dysregulation, and their interaction are associated with maternal anxiety and depressive symptoms at seven months postpartum via negative birth emotions. We conducted four path models to test prenatal self-reported emotion dysregulation and prenatal RSA as separate moderators, as well as stress and fear during childbirth as different mediators. Standardized estimates and standard errors were computed using bias-corrected boot-strapping with 1000 draws. Full information maximum likelihood (FIML) was used to account for unbiased estimates of data. FIML estimators in multiple regression models with missing data are shown to produce less biased parameter estimates, especially compared to listwise deletion, pairwise deletion and mean imputation with the inclusion of appropriate auxiliary variables (Graham, 2012). Little’s Missing Completely at Random (MCAR) test indicated that data likely were MCAR, χ^2^ = 284.00, *p* = .157. To identify appropriate auxiliary variables, we examined if our key variables or demographic variables were associated with attrition. Lower maternal education (χ^2^ = −0.24, *p* = .005) and higher BMI (χ^2^ = 0.05, *p* = .039) were associated with missingness in maternal self-reported internalizing symptoms at 7-months. There were no other significant predictors of missingness in our data. Thus, maternal education and BMI were included as auxiliary variables in all models. We considered birth complications (e.g., use of forceps, unplanned C-sections) as reported in medical records as potential covariates; however, there were no significant associations with our variables of interest similar to other studies (Kaliush et al., 2023a). Therefore, we did not include birth complications as covariates in our models. Model fit was evaluated using *χ*^2^, the comparative fit index (CFI), the Tucker-Lewis index (TLI), standardized root mean square residual (SRMR), and the root mean square error of approximation (RMSEA). Good model fit is indicated by *p* < .05, CFI ≥ .95, TLI ≥ .95, SRMR ≤ .08, RMSEA ≤ .06 (Hu & Bentler, 1999). Data visualizations were created using the *ggplot2* package in R 4.3.2.

## Results

### Descriptive statistics and correlations

Descriptive statistics and correlations for the primary variables are presented in Table 2. There were significant positive associations between childhood maltreatment and prenatal emotion dysregulation (*r* = .19, *p* = .004), birth fear (*r* = .26, *p* = .001), postpartum anxiety problems (*r* = .26, *p* < .001), and postpartum depressive problems (*r* = .24, *p* = .001). Additionally, there were positive associations between prenatal emotion dysregulation and birth fear (*r* = .16, *p* = .045), postpartum anxiety problems (*r* = .48, *p* < .001), and postpartum depressive problems (*r* = .62, *p* < .001). Birth fear was positively associated with birth stress (*r* = .40, *p* < .001) and only anxiety problems postpartum (*r* = .22, *p* = .012). Lastly, greater anxiety problems were associated with greater depressive problems during the postpartum period (*r* = .67, *p* < .001).

### Path models with prenatal DERS as a moderator

**Figure 2.**
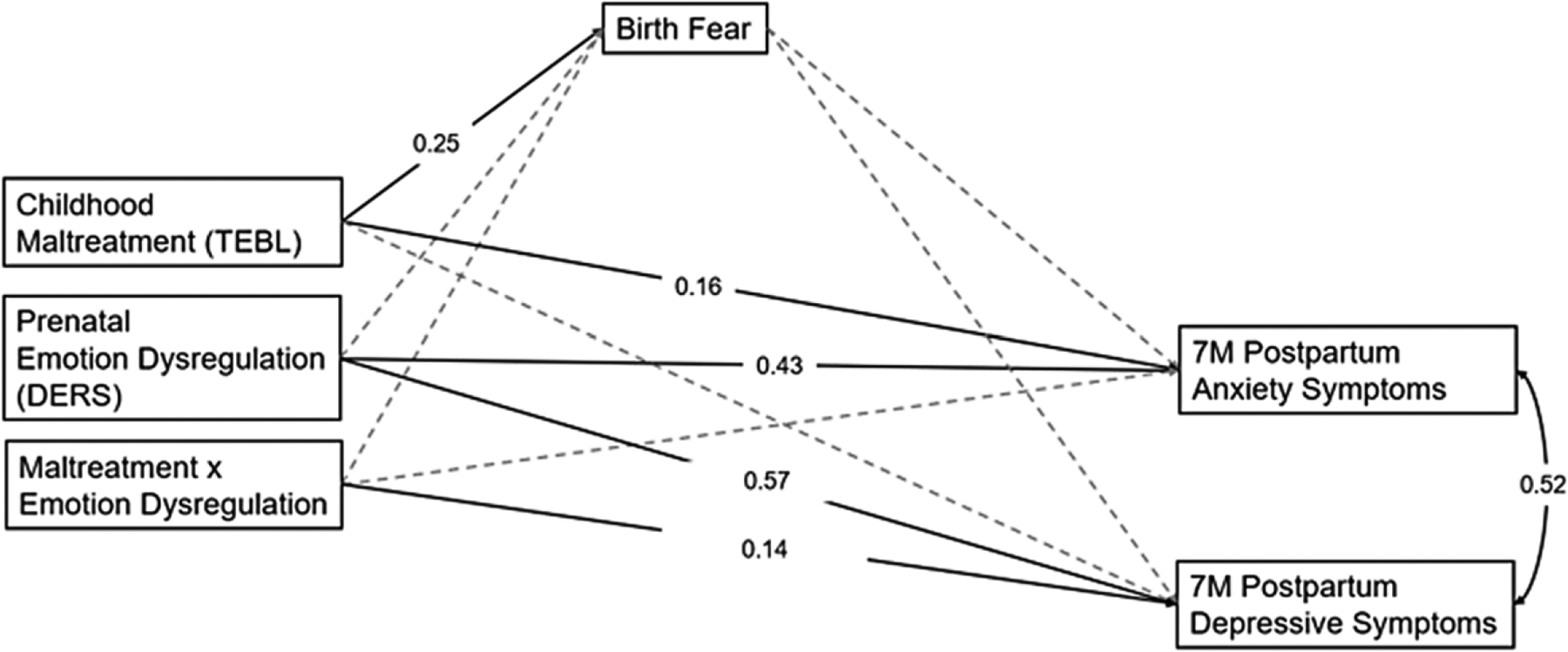
Path model examining the longitudinal relations between childhood maltreatment, prenatal emotion dysregulation (DERS), birth fear, and postpartum anxiety and depressive symptoms.

Figure 2 depicts the path model examining relations between childhood maltreatment accumulation, prenatal maternal emotion dysregulation (DERS), and their interaction on postpartum maternal anxiety and depressive symptoms via fear during birth. The model fit the data well, *χ*^2^ = 2.13 (*p* = .144), CFI = 0.99, TLI = 0.94, RMSEA = 0.08, SRMR = 0.02. Childhood maltreatment was significantly associated with fear during birth (*β* = 0.25, *p* = .004, 95% CI [0.08, 0.41]) as well as postpartum anxiety symptoms (*β* = 0.16, *p* = .018, 95% CI [0.03, 0.28]). Higher prenatal emotion dysregulation was related to greater anxiety (*β* = 0.43, *p* < .001, 95% CI [0.28, 0.56]) and depressive symptoms (*β* = 0.57, *p* < .001, 95% CI [0.46, 0.65]) postpartum.

**Figure 3.**
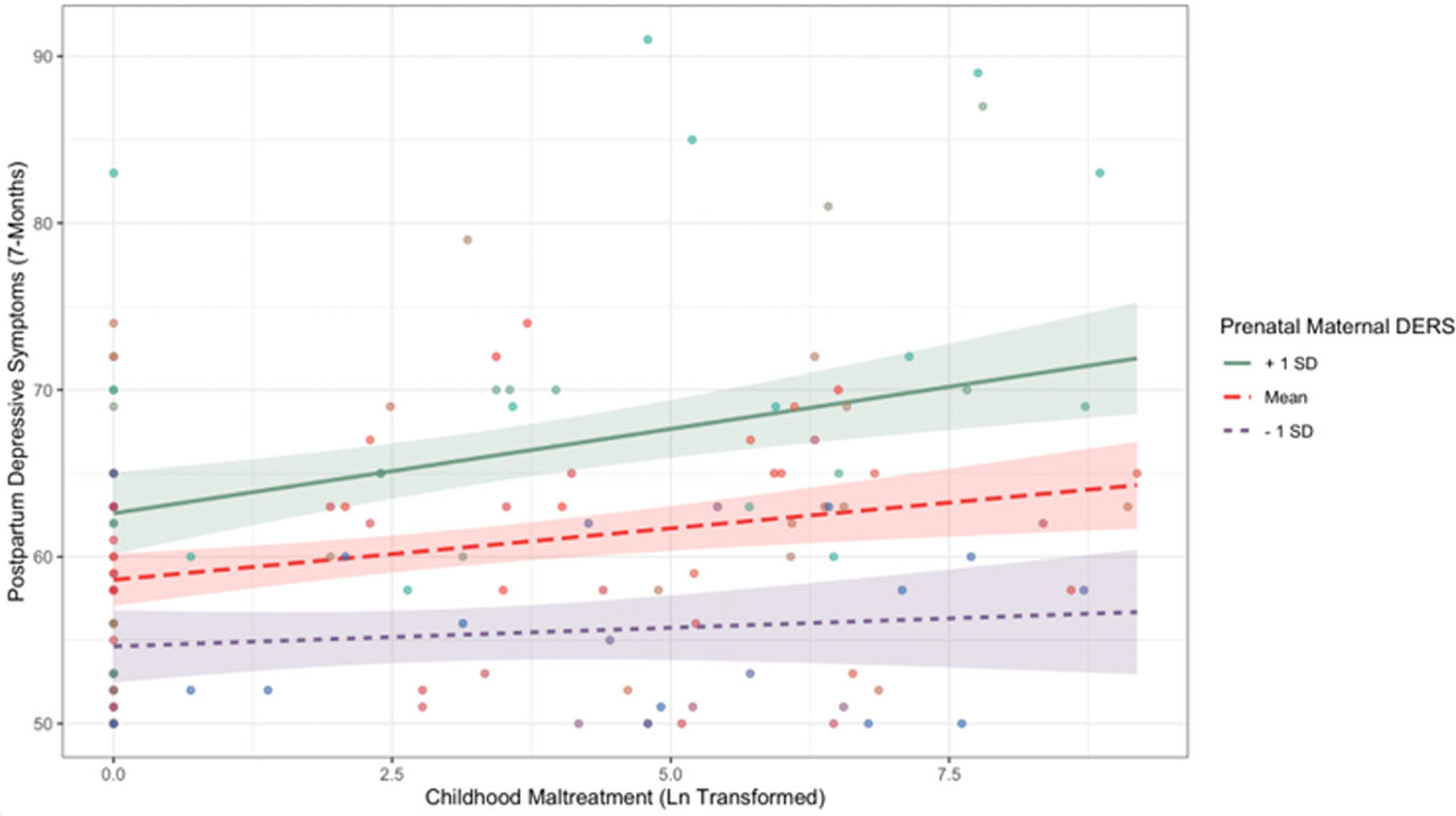
Simple slopes graph depicting the interaction between childhood maltreatment and prenatal emotion dysregulation (DERS) on postpartum depressive symptoms at mean levels of DERS and higher or lower levels of DERS.

Additionally, prenatal emotion dysregulation moderated associations between childhood maltreatment and depressive symptoms (*β* = 0.14, *p* = .025, 95% CI [0.004, 0.25]). Simple slopes testing revealed that there was a significant positive association between childhood maltreatment and postpartum depressive symptoms at mean levels of emotion dysregulation (*b* = 0.62, SE = 0.19, *p* < .001) and higher (1 SD above the mean) levels of emotion dysregulation (*b* = 1.01, SE = 0.27, *p* < .001). Figure 3 depicts the interaction.

**Figure 4.**
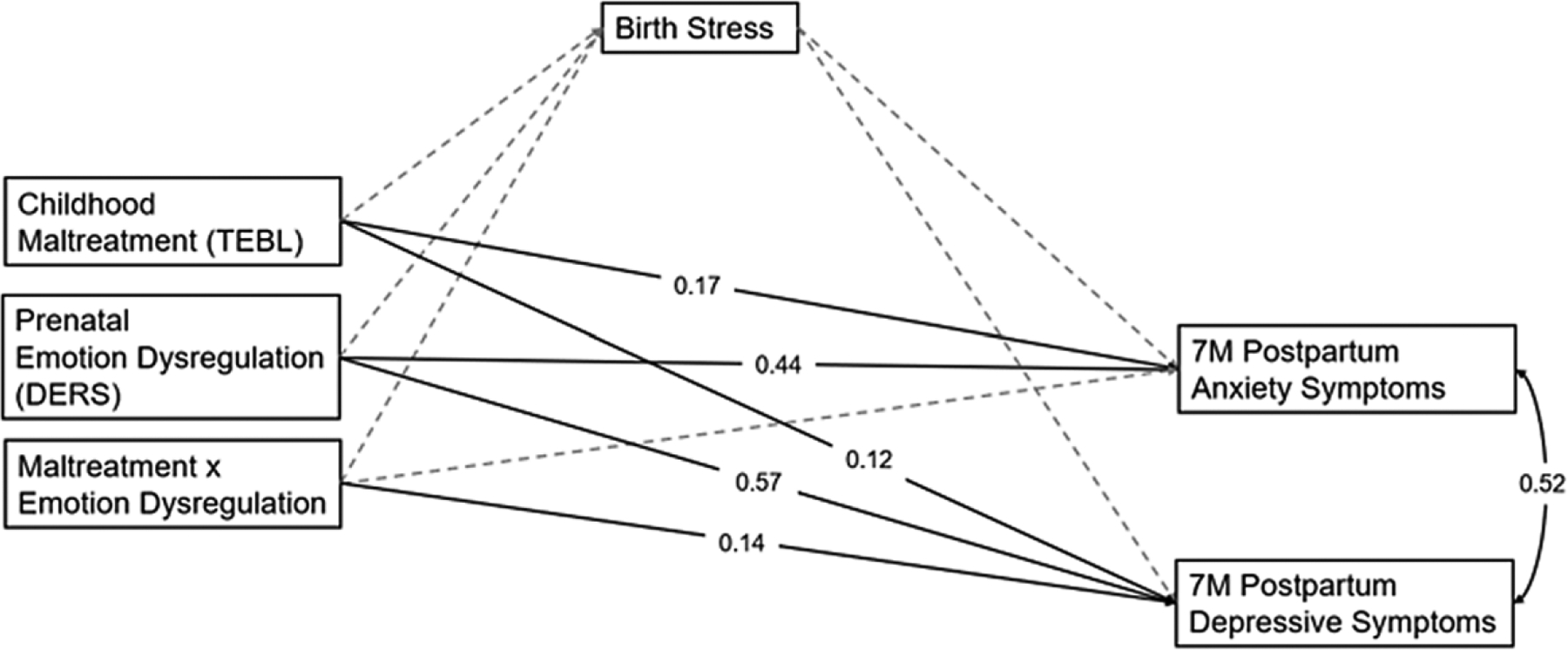
Path model examining the longitudinal relations between childhood maltreatment, prenatal emotion dysregulation, birth stress, and postpartum anxiety and depressive symptoms.

Figure 4 depicts the path model examining relations between childhood maltreatment, prenatal maternal emotion dysregulation, and their interaction on postpartum maternal anxiety and depressive symptoms via stress during birth. The model fit the data well, *χ*^2^ = 1.12 (*p* = .289), CFI = 1.00, TLI = 0.99, RMSEA = 0.03, SRMR = 0.02. Stress was not associated with the other variables in the model. Findings with postpartum anxiety and depressive symptoms were largely consistent with the model with fear during birth. However, in this model, a significant, positive association between childhood maltreatment and postpartum depressive symptoms emerged (*β* = 0.25, *p* = .004, 95% CI [0.08, 0.41]).

### Path models with prenatal maternal RSA as a moderator

**Figure 5.**
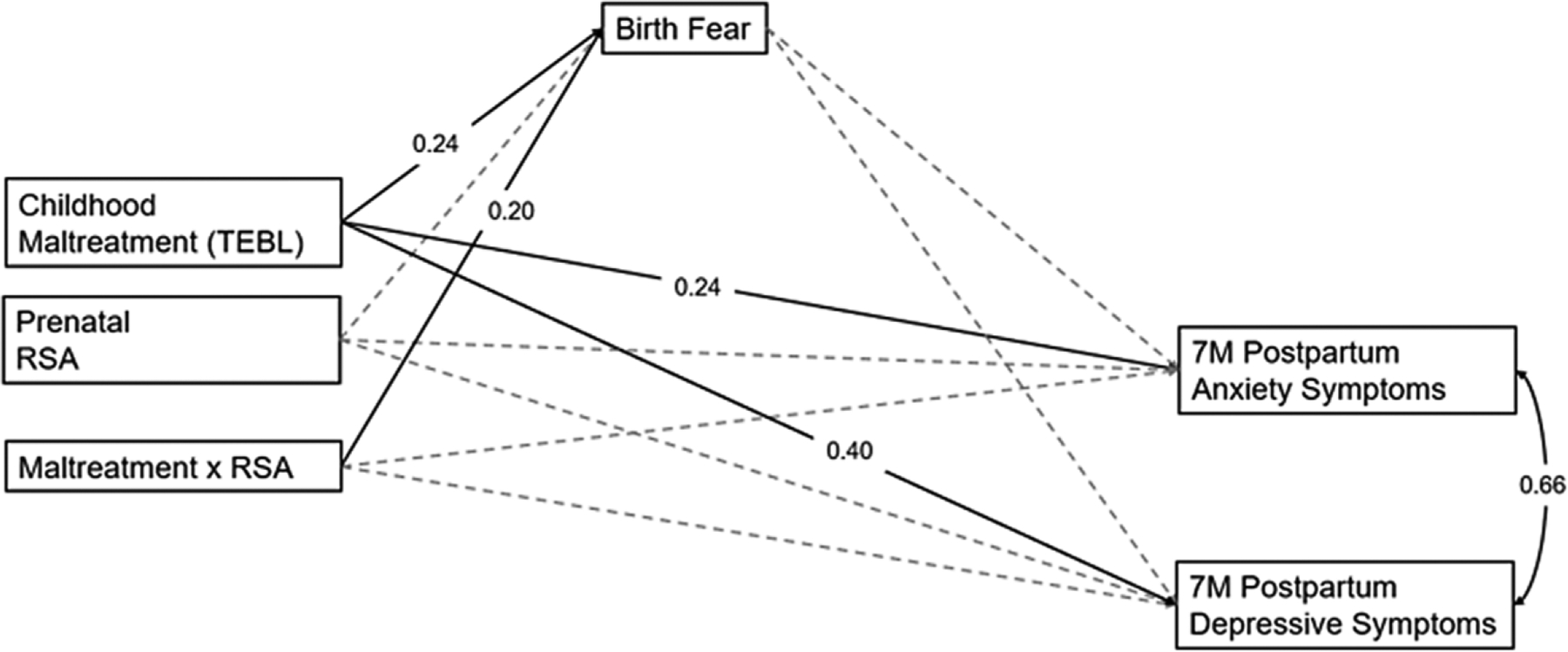
Path model examining the longitudinal relations between childhood maltreatment, prenatal RSA, birth fear, and postpartum anxiety and depressive symptoms.

Figure 5 depicts the path model examining relations between childhood maltreatment accumulation, prenatal maternal RSA, and their interaction on postpartum maternal anxiety and depressive symptoms via fear during birth. The model fit the data well, *χ*^2^ = 0.03 (*p* = .863), CFI = 1.00, TLI = 1.00, RMSEA <0.001, SRMR = 0.003. Childhood maltreatment was significantly associated with fear during birth (*β* = 0.24, *p* = .004, 95% CI [0.07, 0.40]) as well as both postpartum anxiety symptoms (*β* = 0.24, *p* = .005, 95% CI [0.06, 0.39]) and depressive symptoms (*β* = 0.24, *p* = .007, 95% CI [0.07, 0.39]).

**Figure 6.**
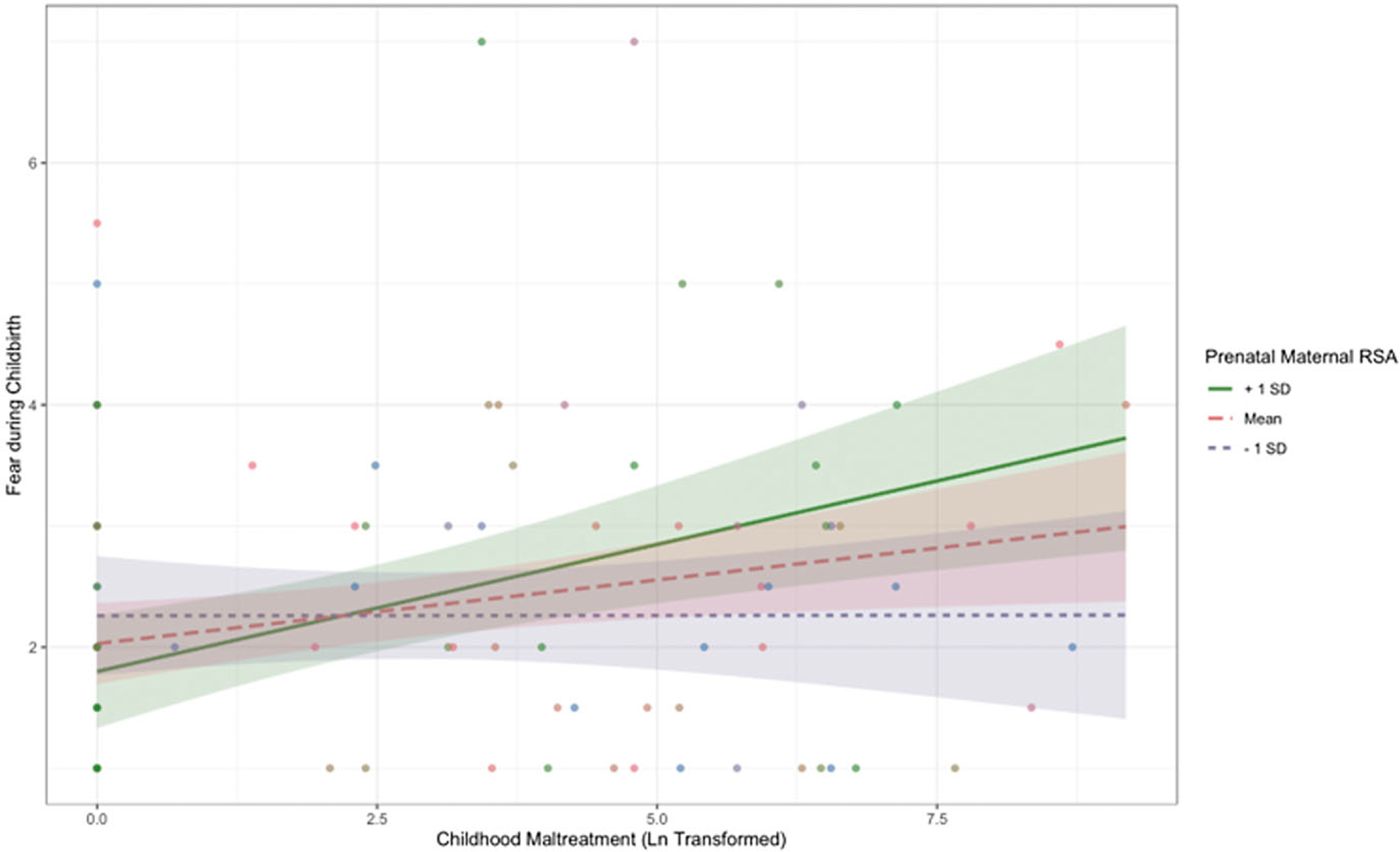
Simple slopes graph depicting the interaction between childhood maltreatment and prenatal emotion dysregulation (RSA) on fear during birth at mean levels of RSA and at lower and higher levels of RSA.

Additionally, prenatal RSA moderated associations between childhood maltreatment and fear during birth (*β* = 0.20, *p* = .020, 95% CI [0.04, 0.37]). Simple slopes testing revealed that there was a significant positive association between childhood maltreatment and fear during birth at mean levels of RSA (*b* = 0.11, SE = 0.04, *p* = .02) and higher (1 SD above the mean) levels of RSA (*b* = 0.21, SE = 0.06, *p* < .001). Figure 6 depicts the interaction.

**Figure 7.**
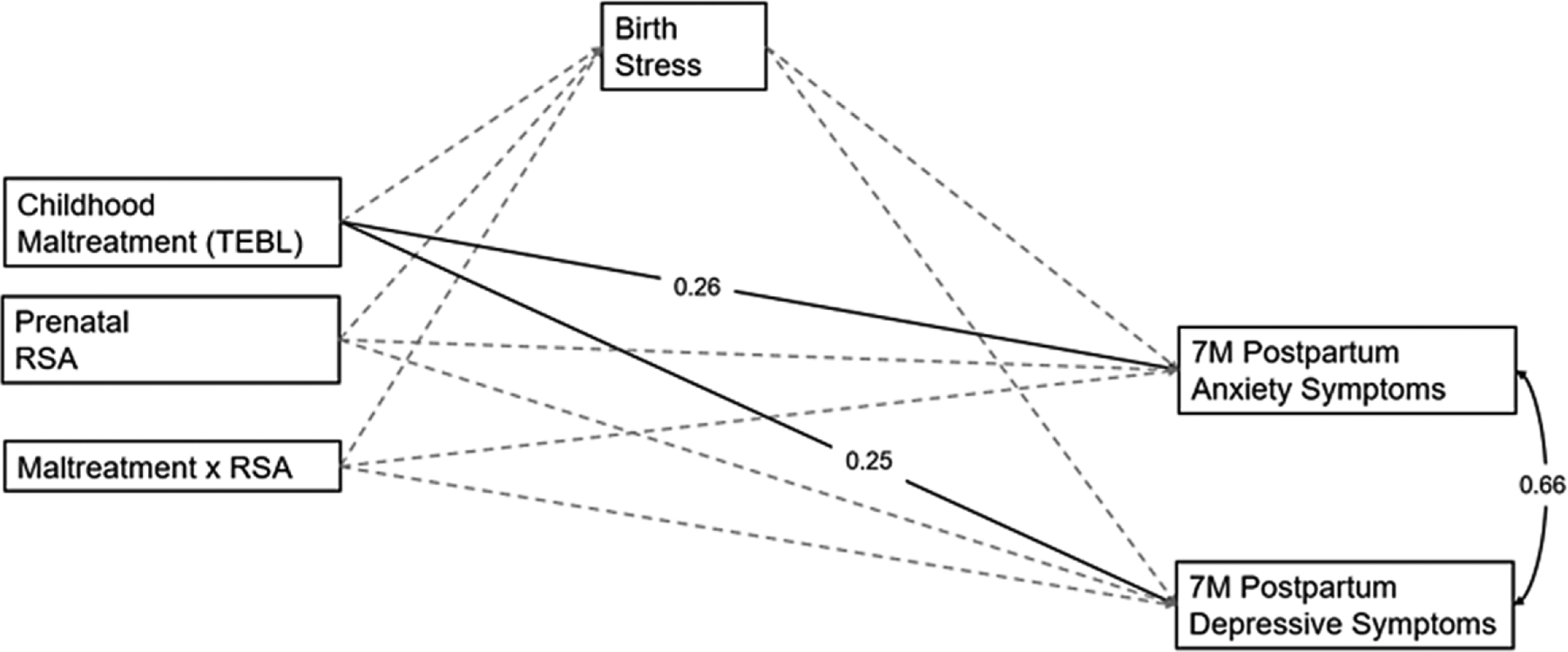
Path model examining the longitudinal relations between childhood maltreatment, prenatal RSA, birth stress, and postpartum anxiety and depressive symptoms.

Figure 7 depicts the path model examining relations between childhood maltreatment accumulation, prenatal maternal RSA, and their interaction on postpartum maternal anxiety and depressive symptoms via stress during birth. The model fit the data well, *χ*^2^ = 5.23 (*p* = .470), CFI = 1.00, TLI = 1.00, RMSEA <0.001, SRMR = 0.01. In this model, there were only significant associations between childhood maltreatment and postpartum anxiety symptoms (*β* = 0.26, *p* = .001, 95% CI [0.09, 0.41]) and depressive symptoms (*β* = 0.25, *p* = .002, 95% CI [0.08, 0.40]).

## Discussion

This study is one of the first to empirically test if negative emotional experiences during birth may be a pathway by which childhood maltreatment is associated with postpartum maternal anxiety and depressive symptoms. In addition, this study examined the moderating role of prenatal emotion dysregulation and physiological regulation. Longitudinal associations between childhood maltreatment, emotion dysregulation, and physiological regulation were examined in association with postpartum outcomes as well. Notably, we found that childhood maltreatment was associated with mothers’ fear for one’s and one’s baby’s life during birth as well as increased symptoms of postpartum anxiety and depressive symptoms. Prenatal emotion dysregulation also predicted higher postpartum depression and anxiety. Prenatal physiological regulation moderated associations between childhood maltreatment and mothers’ fear during birth, such that more childhood maltreatment and higher resting RSA were associated with increased birth fear. Furthermore, prenatal self-reported emotion dysregulation moderated associations between childhood maltreatment and postpartum depressive symptoms, such that more childhood maltreatment and higher emotion dysregulation were associated with increased depressive symptoms at 7 months postpartum. Taken together, these findings underscore the complex, interactive roles of trauma, emotion dysregulation, and physiological responses in influencing postpartum mental health.

### Prenatal RSA moderated associations between childhood maltreatment and fear during birth

A novel finding from our study was that prenatal maternal resting RSA moderated associations between childhood maltreatment and birth fear but not birth stress. Contrary to our hypothesis, higher resting RSA moderated the association between childhood maltreatment history and fear during birth. The interaction between childhood maltreatment and higher prenatal RSA was significantly associated with increased birth fear, suggesting that individuals with childhood maltreatment histories and higher RSA may be experiencing an adaptive response to the birthing process. Higher RSA is often considered a marker of flexibility in emotion regulation, enabling the body to modulate physiological responses effectively under stress (Beauchaine, 2015). In this context, elevated birth fear may not solely indicate vulnerability but could serve as a protective, adaptive response specifically for individuals with childhood maltreatment histories. Previous research shows that individuals who experienced childhood maltreatment demonstrate heightened emotional reactivity to both negative and positive daily events, suggesting a sensitivity to challenges and rewards that may aid in preparedness for major life events (Infurna et al., 2015; McLaughlin et al., 2020). Additionally, previous research has found blunted physiological reactivity among birthing parents with a history of childhood maltreatment (Speck et al., 2024) which has also been associated with maladaptive health outcomes (Bourassa et al., 2021). While we did not examine RSA reactivity in the current study, higher levels of resting RSA among perinatal populations with childhood maltreatment histories may reflect an adaptive sensitivity to stress. Therefore, this may result in heightened awareness and caution toward birth that functions as stress preparation. Future research is needed to address these speculative interpretations. Given the high rates of maternal morbidity in the U.S., fear may be a valid and adaptive response to the real risks and demands of birth (Slomski, 2019).

### Childhood maltreatment, emotion dysregulation, and postpartum mental health

Our findings align with the literature linking childhood maltreatment to postpartum anxiety and depressive symptoms (Fekih-Romdhane et al., 2024; McDonnell & Valentino, 2016; Osofsky et al., 2021). Consistent with the literature, we found strong associations between postpartum anxiety symptoms and postpartum depressive symptoms, serving as an indicator that these symptoms likely co-occur during the postpartum period. Additionally, our study found that prenatal emotion dysregulation was associated with heightened postpartum anxiety and depressive symptoms. This finding emphasizes the role of emotion dysregulation as a transdiagnostic risk factor for internalizing symptoms, consistent with the Research Domain Criteria (RDoC) framework (Cole & Hall, 2008; Cuthbert, 2020). Difficulties in regulating emotions prenatally may increase vulnerability to mental health challenges postpartum (Penner & Rutherford, 2022), especially for mothers with a history of childhood maltreatment. Together, these results underscore the critical need for interventions targeting emotion dysregulation and trauma-related distress during the perinatal period to improve mental health outcomes.

Mother’s self-reported prenatal emotion dysregulation was not associated with negative birth experiences and did not moderate associations between childhood maltreatment and birth fear or stress. However, prenatal emotion dysregulation did moderate associations between childhood maltreatment and postpartum depressive symptoms. Specifically, higher prenatal emotion dysregulation and more childhood maltreatment were associated with higher depressive symptoms at 7 months postpartum. This finding aligns with literature showing that difficulties with emotion regulation in populations with a history of childhood maltreatment are associated with increased mental health difficulties (Dvir et al., 2014). However, this association was not found for postpartum anxiety symptoms in our study. Similar to Choi and Sikkema (2016), anxiety symptoms may be universally increased during the perinatal period and therefore not significantly influenced by trauma histories or negative birth experiences. Additionally, postpartum anxiety symptoms may increase closer to the time of birth, so future research should examine birth experiences and postpartum mental health at various timepoints across the perinatal period.

### Childhood maltreatment, negative birth experiences, and postpartum mental health

Our study supports prior findings that childhood maltreatment is associated with increased fear during birth (Leeners et al., 2016; Lev-Wiesel et al., 2009; Porthan et al., 2023) and this finding was consistent in both models from our study. Among birthing parents with a history of childhood maltreatment, intrusive thoughts and heightened arousal during birth may amplify feelings of fear during birthing processes (Lev-Wiesel et al., 2009). On the other hand, we found no association between childhood maltreatment and *stress* during birth. The measure of fear used in this study captures a more extreme experience during birth, specifically asking if the participant feared for their own or their baby’s life; feeling fear may be more reflective of emotional and regulatory processes compared to the measure of stress in the measures we used. For example, perceiving that the birth experience was out of the birthing parent’s control and did not go as planned may have more to do with factors related to social, medical, and financial resources as well as discriminatory obstetric practices. The measure of stress used addresses these factors such as how much participants felt overwhelmed, if they felt out of control during birth, and if their birth experience went as desired or planned. Therefore, fear and stress during birth are two distinct constructs in this context and may explain different patterns of associations.

### Birth fear and birth stress were not significant mediators during the perinatal period

Contrary to our hypotheses, fear and stress during birth was not a pathway by which childhood maltreatment and prenatal emotion dysregulation were associated with postpartum mental health. First, our finding that birth fear and birth stress were not linked to postpartum mental health outcomes might be explained by characteristics of our sample. We may have had less variability in women’s self-reported negative emotional experiences during birth because pregnant individuals with significant health complications were excluded. Another possible explanation for the null finding between negative birth experiences and postpartum mental health is our use of a validated survey with specific questions about the affective experiences during birth. Studies using idiographic, qualitative methods – where participants describe birth experiences in their own words – have shown contrasting results, suggesting that survey-based measures may miss nuances or do not fully resonate with the full range of participants’ lived experiences (Barnett et al., 2022; Braun et al., 2020).

Additionally, prior research has often focused on limited time points rather than examining earlier risk factors alongside later outcomes (Bell & Andersson, 2016; McKelvin et al., 2021; Simpson & Catling, 2016). For instance, in the present study, birth fear and anxiety symptoms at 7 months postpartum were positively correlated. However, our findings suggest that this correlation may stem from shared prenatal or historical influences, such as childhood maltreatment. It may be that earlier assessments (i.e., historical or prenatal) might reveal more about the origins of this association. Moreover, assessing postpartum mental health only at 7 months postpartum could limit insights into the immediate impact of birth experiences on mental health; birth-related stressors may relate more strongly to outcomes measured closer to birth. It is also possible that while anxiety and depressive symptoms remain relatively stable from pregnancy through the postpartum period (Ahmed et al., 2019; Astbury et al., 2025), other aspects of maternal mental health may be more sensitive to birth experiences. Future research should explore additional time points closer to birth and incorporate qualitative assessments to capture a fuller range of maternal mental health experiences.

### Limitations and future directions

Our findings should be interpreted with our sample in mind and future studies should replicate the design with our limitations in mind. First, excluding individuals with pregnancy complications may have limited the representativeness of our sample and future research should consider looking at a pregnant sample with higher rates of physical complications to expand on the current findings. Although we achieved diversity in socioeconomic status, the sample included limited racial, ethnic, and clinical diversity, particularly among participants with clinically elevated symptoms, which may restrict the generalizability of our findings. Future research should aim for a more inclusive sample, encompassing a broader range of pregnancy, birth experiences, and clinical presentations to improve the applicability and depth of insights into postpartum mental health. The study spanned pre- and post-pandemic years (2019 – 2023), and while we did not examine pandemic-specific factors, birth experiences may have been shaped by contextual influences (e.g., reduced support, infection concerns). These conditions could limit generalizability to post-pandemic births. Additionally, the first assessment of anxiety and depressive symptoms is at 7 months postpartum and perhaps, birth experiences may have more of an impact on mental health assessed closer to birth as parents are beginning to navigate this transition. Future research should address postpartum anxiety and depressive symptoms in relation to negative birth experiences at multiple timepoints during the immediate postpartum period prior to 7 months. Next, our study used only a quantitative measure of subjective birth experiences. Based on the findings in the current study, future research could also use qualitative measures to capture a more comprehensive understanding of birthing experiences and the relation to mental health challenges.

Recruitment methods for the larger longitudinal study did not include individuals with maltreatment histories, and therefore may have resulted in low counts of maltreatment in our sample. However, the rates of maltreatment occurrence in our study are comparable to other studies examining maltreatment in pregnant samples (Atzl et al., 2019). Despite this, future studies should aim to examine associations in a sample with higher rates of maltreatment to further expand on the findings of the current study. Additionally, future studies should consider the timing of maltreatment occurrence as well as different types of maltreatment in association with birth experiences. Lastly, it should be noted that our sample size was determined by power analyses for the specific aims of the larger longitudinal study. Post hoc power analyses were conducted using Monte Carlo simulations, and showed that we have an estimated power of 0.55 to reliably detect our moderation findings between childhood maltreatment and RSA on birth and postnatal outcomes. Future studies with a larger sample size should explore these relations and examine whether moderation findings are replicable and robust.

### Study strengths

This study has several strengths, particularly its longitudinal design, which allowed for data collection across prenatal, birth, and postpartum periods. By examining maternal mental health across these critical time points, our study expands on existing literature that typically focuses on either prenatal-to-birth or birth-to-postpartum transitions. Additionally, we used validated self-report surveys alongside a physiological measure of emotion dysregulation to provide a comprehensive multimethod understanding of factors that contribute to postpartum mental health. Finally, our recruitment strategy achieved a broad distribution of participants with varying levels of emotion dysregulation which increases the inclusivity and relevance of our findings to the broader population.

### Clinical implications

Our findings highlight the value of integrating emotion regulation skills and trauma-informed care into prenatal services to support postpartum mental health, particularly for those with maltreatment histories (Penner & Rutherford, 2022). Programs that teach skills for managing intense emotions, like mindfulness-based cognitive therapy (MBCT; Gu et al., 2015) or dialectical behavior therapy (DBT; Linehan, 1993), could become part of prenatal care to help lower the risk of postpartum mental health challenges (Hellberg et al., 2023). Educating clients about how trauma shapes emotion regulation can help them view their vulnerability as a natural response to past experiences. Framing these challenges as part of a learned history may reduce stigma and self-blame, fostering self-compassion and a sense of control in adopting effective coping techniques (Brähler, 2023).

Additionally, for those experiencing birth fear linked to maltreatment histories, approaches that blend trauma-focused techniques with physiological regulation can help manage stress responses. For example, DBT skills like “TIPP” (Temperature, Intense Exercise, Paced Breathing, Progressive Relaxation) could be integrated into prenatal care to help manage intense arousal and reduce symptoms of anxiety around birth preparation (Linehan, 2014). The “Temperature” skill involves applying cold to the face to activate the mammalian dive response–a reflex that slows the heart rate and reduces physiological arousal. This is one element of TIPP, which offers a suite of effective tools for navigating the emotional and physiological effects of stress, such as those experienced during birth. As many pregnant individuals report feeling disempowered and out of control during labor, equipping them with effective strategies has the potential to foster confidence, resilience, and a sense of agency for individuals during the birthing process (Demirci et al., 2023; McKelvin et al., 2021). Findings from this study suggest that psychoeducation is especially important for individuals who have histories of trauma or are struggling with emotion dysregulation during pregnancy.

## Conclusion

Our study examined birth as a significant psychophysiological event through a developmental psychopathology lens, addressing a critical gap in perinatal research. We found that childhood maltreatment was associated with increased birth fear and heightened postpartum anxiety and depressive symptoms. Notably, prenatal emotion dysregulation moderates the link between childhood maltreatment and postpartum depressive symptoms, suggesting that dysregulation may amplify vulnerability to depressive symptoms and could be a key target for postpartum mental health interventions. We also found that prenatal RSA moderated the association between childhood maltreatment and birth fear, adding to our understanding of the factors influencing maternal experiences during birth. These findings highlight the importance of integrating trauma-informed and emotion-focused care during pregnancy to support postpartum well-being. Future research should explore the mechanisms underlying these associations and evaluate interventions that foster resilience during this reproductive period.

## Supplementary Material

wright 2025 supplemental

**Supplementary material.** The supplementary material for this article can be found athttps://doi.org/10.1017/S0954579425100369.

## Figures and Tables

**Table 1.** Participant demographics

Demographic Variable	*N*	Percent	Mean (SD)	Min-Max
**Maternal Age (years)**	**222**	**99.6%**	**29.62 (4.62)**	**19 – 39**
**Maternal Race and Ethnicity**	**222**	**99.6%**		
**Hispanic/Latina**	**47**	**21.1%**		
American Indian or Alaskan	1	0.4%		
Native				
Black or African American	1	0.4%		
White	24	10.8%		
Self-report race	9	4.0%		
More than 1 race	3	1.3%		
Decline to answer race	9	4.0%		
**Not Hispanic/Latina**	**171**	**76.7%**		
American Indian or Alaskan	1	0.4%		
Native				
Native Hawaiian or Other	5	2.2%		
Pacific Islander				
Asian	11	4.9%		
Black or African American	8	3.6%		
White	132	59.2%		
More than 1 race	13	5.8%		
Decline to answer race	1	0.4%		
**Decline to answer Ethnicity**	**4**	**1.8%**		
White	2	0.9%		
More than 1 race	1	0.4%		
Decline to answer race	1	0.4%		
**Maternal Education**	**222**	**99.6%**		
Less than high school diploma	8	3.6%		
High school graduate or equivalent	24	10.8%		
Some college but did not obtain degree	34	15.2%		
Technical school	13	5.8%		
Obtained associate degree	16	7.2%		
Obtained bachelor degree	71	31.8%		
Obtained Master degree	33	14.8%		
Obtained Doctoral degree	23	10.3%		
**Household Income**	**192**	**86.1%**		
Under $9,000	12	5.4%		
$9,000 – $14,999	3	1.3%		
$15,000 – $19,999	3	1.3%		
$20,000 – $24,999	6	2.7%		
$25,000 – $29,999	9	4.0%		
$30,000 – $39,999	15	6.7%		
$40,000 – $49,999	12	5.4%		
$50,000 – $79,999	49	22.0%		
$80,000 – $99,999	30	13.5%		
$100,000 or more	53	23.8%		
**Relationship Status**	**222**	**99.6%**		
Married	187	83.9%		
Single	8	3.6%		
Partnered	24	10.8%		
Prefer to self-report	1	0.4%		
Separated/Divorced/Widowed	2	0.9%		
**Maternal BMI**	**149**	**66.8%**	**31.91 (7.25)**	**21.14 – 63.67**

*Note*. All demographic data was self-reported and collected at the prenatal timepoint.

**Table 2.** Descriptive table and pearson correlation matrix

	*N*	Mean (SD)	Min-Max	1	2	3	4	5	6	7
*Prenatal*										
1. Childhood Maltreatment	223	2.76 (3.07)	0.00 – 9.44	–						
2. Emotion Dysregulation	223	80.23 (24.26)	36.00 – 165.00	19**	–					
3. Maternal RSA	200	5.56 (1.23)	1.83 – 8.49	−.08	−.01	–				
*Birth*										
1. Birth Fear	155	2.31 (1.35)	1.00 – 7.00	.26**	.16*	−.06	–			
2. Birth Stress	155	4.10 (1.43)	1.20 – 7.00	.07	.14	−.07	.40**	–		
*7-months*										
1. Anxiety Problems	182	5.77 (3.43)	0.00 – 14.00	.26**	.48**	−.05	.22*	.15	–	
2. Depressive Problems	180	7.24 (5.14)	0.00 – 25.00	.24**	.62**	.04	.12	.12	.67**	–

RSA = Respiratory sinus arrhythmia; SD = standard deviation.

* *p* < .05,

** *p* < .01.

## Data Availability

The data that support the findings of this study are available from the corresponding author upon reasonable request.
