# Supplementary material for: A multimethod longitudinal examination of the effects of childhood maltreatment on birth experiences and postpartum mental health
Source: Dev Psychopathol. Author manuscript; Available in PMC 2025 Sep 7. (PMC12414493; doi:10.1017/S0954579425100369)

**Supplemental Materials**

**Table S1**: Items included in Analysis from the Traumatic Experiences of Betrayal Across the Lifespan Measure Subscales

| **Subtypes** | **Measure Items** |
| --- | --- |
| **Physical Abuse** | “*At any time in your life, has someone with whom you were close or trusted seriously hurt you on purpose? Like hitting, pushing, choking, shaking, biting, or burning you? Or punished you so you were badly hurt or bruised?*” |
| **Emotional Abuse** | “*At any time in your life, has someone with whom you were close or trusted called you names, said mean things, or humiliated you to make you feel badly about yourself?*” |
| **Sexual Abuse** | “*At any time in your life, has someone with whom you were close or trusted touched your private sexual body parts when you didn’t want them to or forced you to have some form of sexual contact, such as touching or having sex?*”  “*At any time in your life, has someone other than a close or trusting relationship ever touched your private sexual body parts or forced you to have some form of sexual contact?*” |
| **Physical Neglect** | “*Have you ever had a time in your life when you didn’t have the right care, like not having enough to eat, being homeless, having poor supervision when you were too young, or not being taken to the doctor?*” |
| **Emotional Neglect** | “*At any time in your life, has someone with whom you were close or trusted ignored you or withheld praise or affection to make you feel badly about yourself?*” |

**Table S2: Maltreatment Co-Occurrence Percentages**

| **Number of Maltreatment Subtypes Endorsed** | **Percentage of Participant Endorsements** |
| --- | --- |
| **0** | **48%** |
| **1** | **19%** |
| **2** | **17%** |
| **3** | **12%** |
| **4** | **4%** |
| **5** | **1%** |

**Figure S1**: Bar chart showing the number of participants who endorsed at least one experience within each maltreatment subtype. Overall, 52% (N = 117) reported childhood maltreatment in at least one domain.
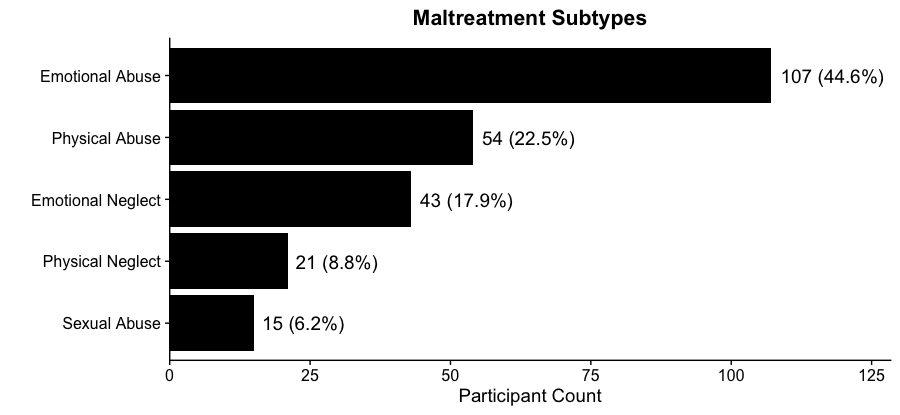

Supplement: wright 2025 supplemental [file NIHMS2103870-supplement-wright_2025_supplemental.docx]
